# The Evaluation of Effective Drugs for the Treatment of Non-Alcoholic Fatty Liver Disease: A Systematic Review and Network Meta-Analysis

**DOI:** 10.34172/apb.2020.065

**Published:** 2020-08-09

**Authors:** Ramin Jalili, Mohammad Hossein Somi, Hossein Hosseinifard, Fatemeh Salehnia, Morteza Ghojazadeh, Nima Makhdami, Masoud Shirmohammadi

**Affiliations:** ^1^Department of Internal Medicine, Tabriz University of Medical Sciences, Tabriz, Iran.; ^2^Liver and Gastrointestinal Diseases Research Center, Tabriz University of Medical Sciences, Tabriz, Iran.; ^3^Research Center for Evidence Based Medicine (RCEBM), Tabriz University of Medical Sciences, Tabriz, Iran.; ^4^St Joseph’s Hospital, Hamilton, Canada.; ^5^Department of Gastroenterology, Liver, and Gastrointestinal Diseases Research Center, Tabriz University of Medical Sciences, Tabriz, Iran.

**Keywords:** Non-alcoholic fatty liver disease, Therapeutic, Systematic review, Network meta-analysis

## Abstract

***Purpose:*** Non-alcoholic fatty liver disease (NAFLD) and steatohepatitis are two forms of fatty liver disease with benign and malignant nature, respectively. These two conditions can cause an increased risk of liver cirrhosis and hepatocellular carcinoma. Given the importance and high prevalence of NAFLD, it is necessary to investigate the results of different studies in related scope to provide a clarity guarantee of effectiveness. Therefore, this systematic review and meta-analysis aim to study the efficacy of various medications used in the treatment of NAFLD.

***Methods:*** A systematic search of medical databases identified 1963 articles. After exclusion of duplicated articles and those which did not meet our inclusion criteria, eta-analysis was performed on 84 articles. Serum levels of alanine aminotransferase (ALT), aspartate amino transferase (AST) were set as primary outcomes and body mass index (BMI), hepatic steatosis, and NAFLD activity score (NAS) were determined as secondary outcomes.

***Results:*** Based on the P-score of the therapeutic effects on the non-alcoholic steatohepatitis (NASH), we observed the highest efficacy for atorvastatin, tryptophan, orlistat, omega-3 and obeticholic acid for reduction of ALT, AST, BMI, steatosis and NAS respectively.

***Conclusion:*** This meta-analysis showed that atorvastatin. life-style modification, weight loss, and BMI reduction had a remarkable effect on NAFLD-patients by decreasing aminotransferases.

## Introduction

The non-alcoholic fatty liver disease (NAFLD) covers a broad spectrum of liver disease ranging from pure fatty liver to non-alcoholic steatohepatitis (NASH) and finally cirrhosis. It occurs in patients without a significant alcohol intake.^1^ Although the pathogenesis of NAFLD is not well-understood, some studies support the “two-hit theory.” While the “first hit” involves insulin resistance and results in the collection of fat in the liver, the “second hit” consists of oxidative stress resulting in lipid peroxidation, hepatocellular degeneration, cell death, hepatic stellate cell (HSC) activation, and fibrogenesis.^2,3^

End-stage liver disease and hepatocellular carcinoma secondary to NAFLD are the second leading cause of liver transplantation in the United States. This is an alarming trend that NAFLD is replacing hepatitis C as the most common indication for liver transplantation during the future decades.^4-6^

As a result of obesity epidemics, the prevalence of NAFLD, as a common liver disease, has dramatically increased. Estimations reveal an incidence of up to 20%-30% for NAFLD in Western and 5-18% in Asian countries. ^7^ Due to the inappropriate diet and sedentary lifestyle, the increased prevalence of NAFLD throughout the world is considered a common clinical concern over time.^8^

Weight loss, achieved either by a hypocaloric diet alone or in combination with increased physical activity, reduces hepatic steatosis a combination of a hypocaloric diet (daily caloric reduction of 500-1000 kcal), and moderate-intensity exercise is presumably to provide the best likelihood of improving steatosis. However, a more significant weight loss (7%-10%) is required to improve the majority of the histopathological features of NASH.^9^

Although NAFLD is associated with components of metabolic syndrome, the presence of an increasing number of metabolic disorders, including insulin resistance, type 2 diabetes, hypertension dyslipidemia, and visceral obesity, seems to increase the risk of progressive fatty liver disease.^10-12^ Therefore, NAFLD-patients are at the highest risk for adverse outcomes because of multiple risk factors such as type-2 diabetes mellitus (T2DM) and hypertension.^13^

The current standard of care (SOC) for managing NAFLD mainly includes caloric restriction of 25–30 kcal/kg/day ideal body weight and moderate physical activity.^14^

Although there are no well-established guidelines recommendations for managing NAFLD,^15^ medications that are mainly prescribed include antioxidants (e.g. vitamins E and C, betaine), insulin-sensitizing agents (thiazolidinediones and metformin), lipid-lowering agents (statins, orlistat, probucol), choleretic agents such as ursodeoxycholic acid (UCDA), and medications with anti-inflammatory (pentoxifylline) or anti-fibrotic (angiotensin-receptor blockers) potential.^16^

Given the enormous variety of prescribed drugs in clinical trials for the treatment of NAFLD, multi-drug combination studies and many not-investigated drugs in head-to-head trials, we have aimed to evaluate the effectiveness of most commonly used medications on reducing liver transaminases (alanine aminotransferase (ALT), aspartate amino transferase (AST), body mass index (BMI), NAFLD activity score (NAS) and steatosis in patients with NAFLD in a systematic review and network meta-analysis.

## Materials and Methods

### Search methodology

This systematic review and network meta-analysis of randomized clinical trials evaluate the efficacy of NAFLD treatments. Medline, Scopus, Web of Science, Embase, and Cochran Library databases were searched for clinical trials that examined the effects of therapeutic interventions in NAFLD patients. We used the following keywords in our database search: NAFLD, non-alcoholic fatty liver disease, fatty liver, fatty liver disease, NASH, hepatic steatosis, and steatohepatitis by combining the OR, AND and NOT operators. Table 1, illustrates the abbreviations that are used throughout this paper to describe the different studied medications.

**Table 1:** Medication name and their abbreviations used in this manuscript

**Medication name**	**Abbreviation**	**Medication name**	**Abbreviation**
Metformin	Met	Ipragliflozin	Lpra
Melatonin	Mela	Losartan	Losa
Liraglutide	Lira	Insulin	Insu
L-carnitine	L-Car	Gliclazide	Glicl
Sitagliptin	Sita	Rosiglitazone	Rosi
Silymarin	Silly	Resveratrol	Resv
Probucol	Probu	Pentoxifylline	Pant
Probiotic	Prob	Orlistat	Orli
Placebo	Plac	Omega-3 Acid	Omeg3
Pioglitazone	Piog	Obeticholic	Obet
Fenofibrate	Feno	Exenatide	Exen
Ezetimbe	Ezet	Colesevelam	Coles
Bicycline	Bicy	Atorvastatin	Atro
Betaine	Beta	Vitamin- E	Vit-E
Anti-Oxidants	Antiox	Vildagliptin	Vida
Ursodeoxycholic acid	UDCA	Tocotrienol	Toco
Tryptophan	Tryp	Telmisartan	Telm
Symbiotic	Synb	Standard of Care	SOC

Manual search also performed to prevent the loss of published articles in the study sources. Correspondence was communicated with the authors of selected articles in order to obtain unpublished articles as well.

### Inclusion, exclusion and eligibility criteria

We included publications that: had studied the various medical interventions for the treatment of NAFLD, were published in English language, were published to January 2019 and also paperers which were presented in different conferences.

We excluded: articles that were published in languages other than English, animal studies, research studies with no apparent sample size and, low quality research works.

Additionally, we only included clinical trials that were conducted on patients with NAFLD and had compared an active drug with another active drug or placebo.

## Methodological quality

### Select studies

The selected articles were processed in 3 states by the subject experts: The titles of all review and incompatible articles were excluded. Abstracts and full texts of the articles were studied.

The selected studies were evaluated by the two experts for recognizing the risk of bias using Joanna Briggs Institute tool for critically appraising the clinical trials; the controversies between them were resolved by a third expert.^17^ The critical appraisal form of the select studies is shown in Table S1.^18-101^

The extracted information was summarized in the data extraction table (Table 2). Extracted data were included: first author, publication year, country, sample size, and number of participants over. Finally, the Endnote X5 software was used to manage the references.

**Table 2:** Critical appraisal results of eligible studies

**Study**	**Q1**	**Q2**	**Q3**	**Q4**	**Q5**	**Q6**	**Q7**	**Q8**	**Q9**	**Q10**	**Q11**	**Q12**	**Q13**
Marchesini et al (2001)^18^	Y	N	Y	Y	Y	U	Y	Y	Y	Y	Y	Y	Y
Athyros et al (2006)^19^	Y	N	Y	N	N	U	Y	Y	Y	Y	Y	Y	Y
Ratziu et al (2008)^20^	Y	N	Y	Y	Y	N	Y	Y	Y	Y	Y	Y	Y
Hatzitolios et al (2004)^21^	Y	N	Y	Y	Y	U	Y	Y	Y	Y	Y	Y	Y
Fan et al (2013)^22^	Y	N	Y	N	N	N	Y	Y	Y	Y	Y	Y	Y
Capanni et al (2006)^23^	Y	N	Y	Y	Y	N	Y	Y	Y	Y	Y	Y	Y
Solhi et al (2014)^24^	Y	N	U	U	U	U	Y	Y	Y	Y	Y	Y	Y
Khoshbaten et al (2010)^25^	Y	N	Y	Y	N	N	Y	Y	Y	Y	Y	Y	Y
Lindor et al (2004)^26^	Y	N	Y	Y	Y	Y	Y	Y	Y	Y	Y	Y	Y
Zein et al (2011) ^27^	Y	Y	Y	Y	Y	Y	Y	Y	Y	Y	Y	Y	Y
Mumtaz et al (2017)^28^	Y	N	Y	Y	Y	U	Y	Y	Y	Y	Y	Y	Y
Shahebrahimi et al (2017)^29^	Y	N	Y	Y	N	N	Y	Y	Y	Y	Y	Y	Y
Wong et al (2017) ^30^	Y	N	Y	Y	Y	Y	Y	Y	Y	Y	Y	Y	Y
Oscarsson et al (2018)^31^	Y	N	Y	Y	Y	U	Y	Y	Y	Y	Y	Y	Y
Ito et al (2017)^32^	Y	N	Y	N	N	N	Y	Y	Y	Y	Y	Y	Y
Asghari et al (2018)^33^	Y	Y	Y	Y	Y	Y	Y	Y	Y	Y	Y	Y	Y
Celinski et al (2014)^34^	Y	N	Y	U	U	U	Y	Y	Y	Y	Y	Y	Y
Neuschwander-Tetri et al (2015)^35^	Y	Y	Y	Y	Y	N	Y	Y	Y	Y	Y	Y	Y
Abdelmalek et al (2009)^36^	Y	Y	Y	Y	Y	Y	Y	Y	Y	Y	Y	Y	Y
Alam et al (2016) ^37^	Y	N	Y	N	N	N	Y	Y	Y	Y	Y	Y	Y
Aller et al (2011)^38^	Y	N	Y	Y	Y	U	Y	Y	Y	Y	Y	Y	Y
Lonardo et al (2015)^39^	Y	N	Y	Y	Y	Y	Y	Y	Y	Y	Y	Y	Y
Armstrong et al (2016)^40^	Y	N	Y	Y	Y	Y	Y	Y	Y	Y	Y	Y	Y
Balas et al (2007)^41^	Y	Y	Y	Y	Y	Y	Y	Y	Y	Y	Y	Y	Y
Baniasadi et al (2015)^42^	Y	N	Y	Y	Y	N	Y	Y	Y	Y	Y	Y	Y
Belfort et al (2006)^43^	Y	N	Y	Y	Y	N	Y	Y	Y	Y	Y	Y	Y
Chachay et al (2014)^44^	Y	N	Y	Y	Y	Y	Y	Y	Y	Y	Y	Y	Y
Xu et al (2015)^45^	Y	N	Y	Y	Y	Y	Y	Y	Y	Y	Y	Y	Y
Cui et al (2016)^46^	Y	Y	Y	Y	Y	Y	Y	Y	Y	Y	Y	Y	Y
Cusi et al (2016)^47^	Y	N	Y	Y	Y	Y	Y	Y	Y	Y	Y	Y	Y
Deng et al (2017)^48^	Y	Y	Y	Y	Y	Y	Y	Y	Y	Y	Y	Y	Y
Ebrahimi et al (2016)^49^	Y	N	Y	Y	Y	N	Y	Y	Y	Y	Y	Y	Y
Ekhlasi et al (2017)^50^	Y	Y	Y	Y	Y	Y	Y	Y	Y	Y	Y	Y	Y
Eslamparast et al (2014)^51^	Y	Y	Y	Y	Y	Y	Y	Y	Y	Y	Y	Y	Y
Faghihzadeh et al (2014)^52^	Y	N	Y	Y	Y	Y	Y	Y	Y	Y	Y	Y	Y
Feng et al (2017)^53^	Y	N	Y	Y	Y	Y	Y	Y	Y	Y	Y	Y	Y
Garinis et al (2010) ^54^	Y	N	Y	Y	Y	Y	Y	Y	Y	Y	Y	Y	Y
Hajiaghamohammadi et al (2012)^55^	Y	N	Y	U	U	U	Y	Y	Y	Y	Y	Y	Y
Han et al (2014)^56^	Y	N	Y	U	U	U	Y	Y	Y	Y	Y	Y	Y
Hannah and Harrison (2003)^57^	Y	Y	Y	Y	Y	Y	Y	Y	Y	Y	Y	Y	Y
Harrison et al (2009)^58^	Y	N	Y	Y	Y	U	Y	Y	Y	Y	Y	Y	Y
Haukeland (2009)^59^	Y	Y	Y	Y	Y	Y	Y	Y	Y	Y	Y	Y	Y
Heebøll et al (2016)^60^	Y	Y	Y	Y	Y	Y	Y	Y	Y	Y	Y	Y	Y
Hirataet al (2013)^61^	Y	N	Y	N	N	N	Y	Y	Y	Y	Y	Y	Y
Hussain et al (2016)^62^	Y	N	Y	U	U	U	Y	Y	Y	Y	Y	Y	Y
Khoo et al (2017)^63^	Y	N	Y	Y	Y	U	Y	Y	Y	Y	Y	Y	Y
Le et al (2012)^64^	Y	Y	Y	Y	Y	Y	Y	Y	Y	Y	Y	Y	Y
Lee et al (2008)^65^	Y	Y	Y	Y	Y	U	Y	Y	Y	Y	Y	Y	Y
Liechti et al (2012)^66^	Y	N	Y	Y	Y	N	Y	Y	Y	Y	Y	Y	Y
Le and Wang (2017)^67^	Y	N	Y	N	N	Y	Y	Y	Y	Y	Y	Y	Y
Loomba (2015)^68^	Y	Y	Y	Y	Y	N	Y	Y	Y	Y	Y	Y	Y
McPherson et al (2017)^69^	Y	Y	Y	Y	Y	Y	Y	Y	Y	Y	Y	Y	Y
Mendez-Sanchez et al (2004)^70^	Y	N	Y	Y	Y	U	Y	Y	Y	Y	Y	Y	Y
Merat et al (2003)^71^	Y	Y	Y	Y	Y	Y	Y	Y	Y	Y	Y	Y	Y
Mofidi et al (2017)^72^	Y	N	Y	Y	Y	U	Y	Y	Y	Y	Y	Y	Y
van Wagner et al (2011)^73^	Y	N	Y	Y	Y	Y	Y	Y	Y	Y	Y	Y	Y
Dagan et al (2006) ^74^	Y	Y	Y	Y	Y	Y	Y	Y	Y	Y	Y	Y	Y
Wah Kheong et al (2017)^75^	Y	Y	Y	Y	Y	Y	Y	Y	Y	Y	Y	Y	Y
Wong et al (2013)^76^	Y	N	Y	U	U	Y	Y	Y	Y	Y	Y	Y	Y
Tiikkainen et al (2004)^77^	Y	N	Y	Y	Y	U	Y	Y	Y	Y	Y	Y	Y
Zhu et al (2008)^78^	Y	N	Y	U	U	Y	Y	Y	Y	Y	Y	Y	Y
Mudaliar et al (2013)^79^	Y	Y	Y	Y	Y	U	Y	Y	Y	Y	Y	Y	Y
Nelson et al (2009)^80^	Y	Y	Y	Y	Y	Y	Y	Y	Y	Y	Y	Y	Y
Nogueira et al (2016)^81^	Y	N	Y	Y	Y	Y	Y	Y	Y	Y	Y	Y	Y
Omer et al (2009-10)^82^	Y	N	Y	N	N	Y	Y	Y	Y	Y	Y	Y	Y
Pakravan et al (2017)^83^	Y	N	Y	Y	Y	U	Y	Y	Y	Y	Y	Y	Y
Parikh et al (2016)^84^	Y	N	Y	Y	Y	U	Y	Y	Y	Y	Y	Y	Y
Ratziu et al (2016)^85^	Y	N	Y	Y	Y	Y	Y	Y	Y	Y	Y	Y	Y
Shenoy et al (2014) ^96^	Y	N	Y	Y	Y	Y	Y	Y	Y	Y	Y	Y	Y
Shang et al (2008)^95^	Y	N	Y	Y	Y	U	Y	Y	Y	Y	Y	Y	Y
Sofi et al (2010)^94^	Y	N	Y	U	U	U	Y	Y	Y	Y	Y	Y	Y
Takeshita et al (2014)^93^	Y	Y	Y	Y	Y	Y	Y	Y	Y	Y	Y	Y	Y
Shibuya et al (2017)^92^	Y	N	Y	Y	Y	U	Y	Y	Y	Y	Y	Y	Y
Chen et al (2015)^91^	Y	N	Y	Y	Y	Y	Y	Y	Y	Y	Y	Y	Y
Sofer (2011)^100^	Y	N	Y	Y	Y	U	Y	Y	Y	Y	Y	Y	Y
Sharma et al (2012)^90^	Y	N	Y	N	N	Y	Y	Y	Y	Y	Y	Y	Y
Harrison et al (2014)^89^	Y	N	Y	Y	Y	U	Y	Y	Y	Y	Y	Y	Y
Sanyal et al (2010)^88^	Y	Y	Y	Y	Y	Y	Y	Y	Y	N	Y	Y	Y
Santos et al (2003)^87^	Y	N	U	Y	Y	N	Y	Y	Y	Y	Y	Y	Y
Razavizade et al (2013)^86^	Y	Y	Y	Y	Y	Y	Y	Y	Y	Y	Y	Y	Y
Hameed et al (2017)^97^	Y	Y	Y	Y	Y	Y	Y	Y	Y	Y	Y	Y	Y
Hosseinpour-Arjmand et al (2018)^98^	Y	Y	Y	Y	Y	Y	Y	Y	Y	Y	Y	Y	Y
Kuchay et al (2018)^99^	Y	N	U	N	N	Y	Y	Y	Y	Y	Y	Y	Y
Abenavoli et al (2017)^101^	Y	N	Y	Y	Y	U	Y	Y	Y	Y	Y	Y	Y
Total %	100	30.9	96.4	79.7	77.3	51.1	100	100	100	100	100	100	100

Y = Yes, N = No, U = Unclear; JBI critical appraisal checklist for randomized controlled trials: Q1 = Was true randomization used for assignment of participants to treatment groups?; Q2 = Was allocation to treatment groups concealed?; Q3 = Were treatment groups similar at baseline?; Q4 = Were participants blind to treatment assignment?; Q5 = Were those delivering treatment blind to treatment assignment?; Q6 = Were outcome assessors blind to treatment assignment?; Q7 = Were treatment groups treated identically other than the intervention of interest?; Q8 = Was follow-up complete, and if not, were strategies to address incomplete follow-up utilized?; Q9 = Were participants analyzed in the groups to which they were randomized?; Q10 = Were outcomes measured in the same way for treatment groups?; Q11 = Were outcomes measured in a reliable way?; Q12 = Was appropriate statistical analysis used?; Q13 = Was the trial design appropriate, and any deviations from the standard RCT design (individual randomization, parallel groups) accounted for in the conduct and analysis of the trial?

### Publication bias

**Figure 1:**
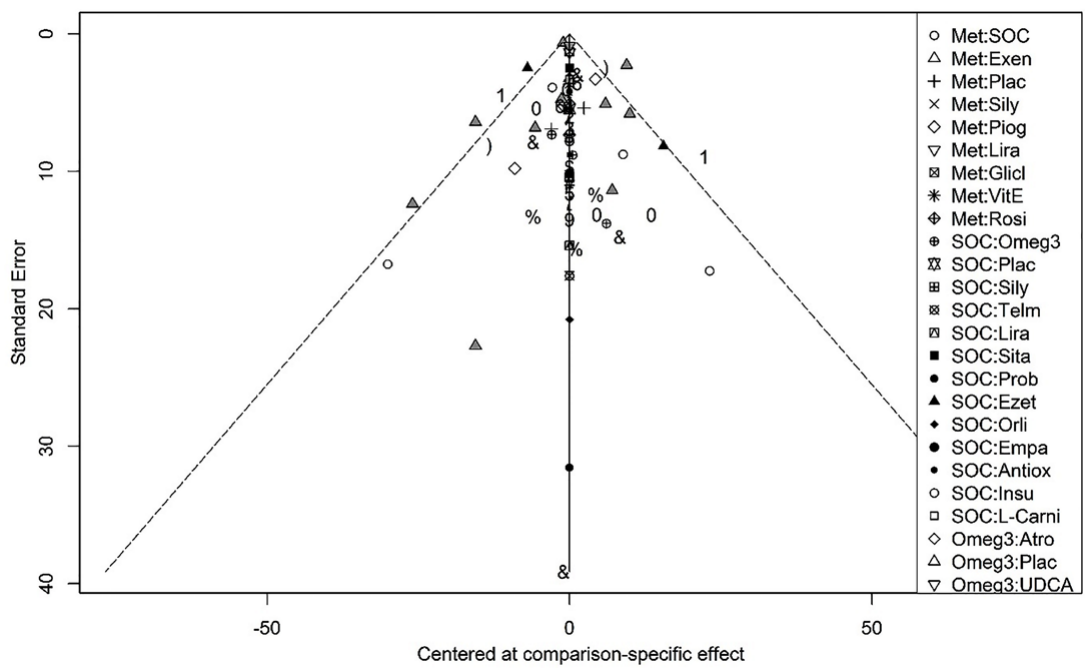
Funnel plot of selected articles showing degrees of publication bias.

The funnel plot (Figure 1) illustrates the publication bias of the studies that have been entered into the network meta-analysis. This symmetry shows that there has been evidence of publication bias in this meta-analysis.

### Primary and secondary outcomes in the study

The primary outcome measure in this study was the mean changes in ALT and AST levels.

Changes in BMI, NAS, and steatosis were considered as secondary outcomes.

### Statistical methods

Investigation of stability and similarity assumptions in intervention network:

To assess the similarity hypothesis, the baseline characteristics of the participants were assessed, and trials with no similarity hypothesis were included in the network meta-analysis.

The heterogeneity between studies was analyzed via the Cochran Q test and I-square statistic.

Rankings of treatments P-scores were used to rank for each treatment. P-scores are calculated on the basis of defect estimation and standard error estimates in the grid. The wake score is between zero and one, and the closer the wake of a cure to a cure, the better the cure. We have utilized the GRADE approach to calculate the level of evidence; the level of evidence has been categorized to very low, low, moderate and high taking bias, inconsistency, indirectness and imprecision into account.

## Results and Discussion

Search results and basic characteristics of studies entered into a meta-analysis of networks. The data extraction is represented in Table S1.

**Figure 2:**
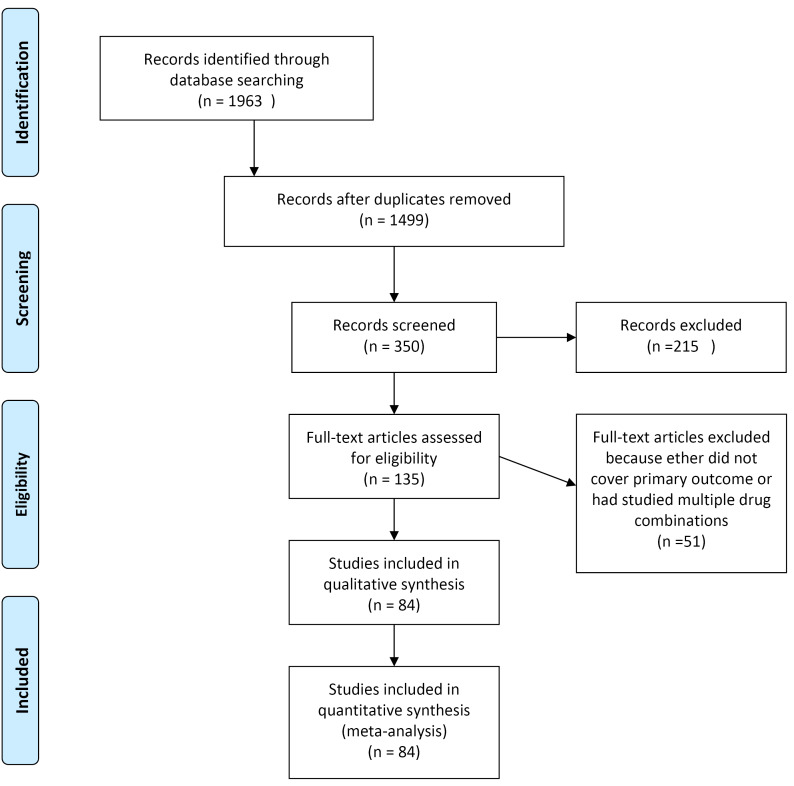
PRISMA flow diagram.

After excluding duplicate papers, and papers that did not meet the inclusion criteria we entered 84 to the final meta-analysis. PRISMA (Preferred Reporting Items for Systematic Reviews and Meta-Analyses) diagram (Figure 2) illustrates the number of the articles screened for final number of analyzed articles.

### Network meta-analysis results for primary outcomes

#### Comparison of the effects of treatments on ALT

**Figure 3:**
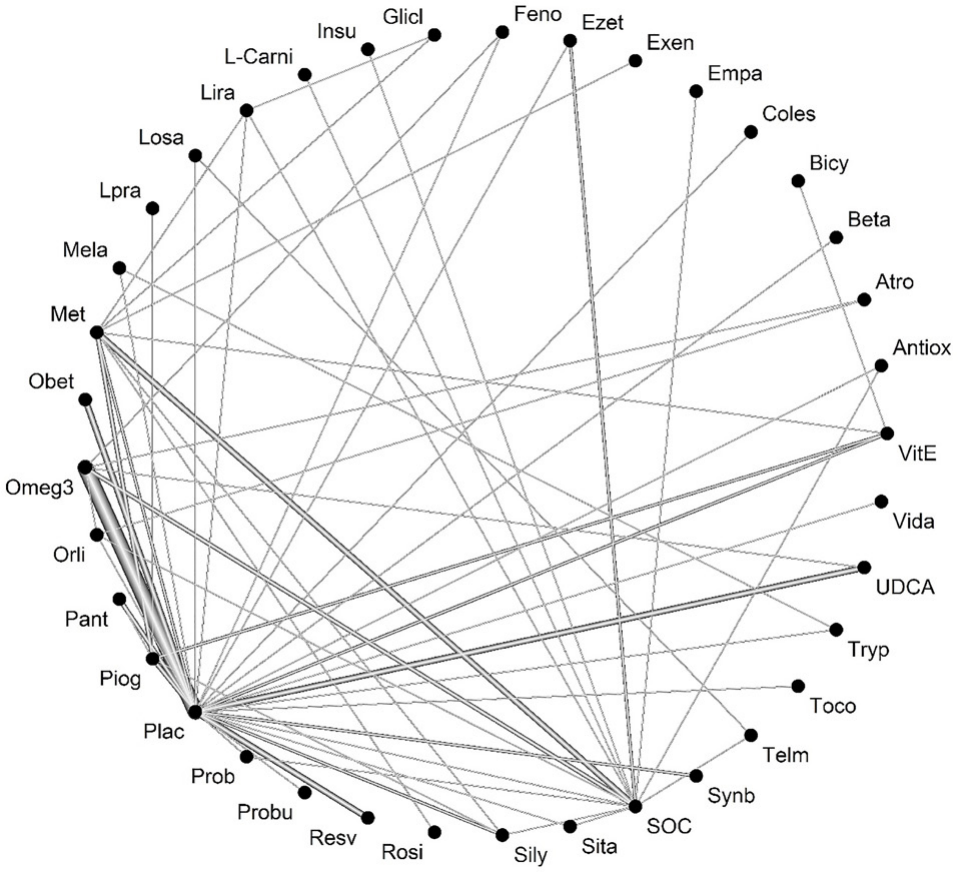
Network of interventions introduced into the network meta-analysis for ALT outcome.
Legend: Feno: fenofibrate, Ezet: ezetimbe, Exen: exenatide, Coles: Colesevelam, Bicy: bicycline, Beta: betaine, Atrp: atrovastatin, Antiox: anti-oxidants, Vit-E: vitamin- E, Vida:vidagliptin, UDCA: Ursodeoxycholic acid, Tryp: tryptophan, Toco: Tocotrienol, Telm: telmisartan, Synb:symbiotic, SOC: Standard of Care, Sita: sitagliptin, Silly:silymarin, Rosi: rosiglitazone, Resv: Resveratrol, Probu: probucol, Prob: probiotic, Plac:placebo, Piog: pioglitazone, Pant: Pentoxifylline, Orli: Orlistat, Omeg3: Omega-3, Obet: obeticholic acid, Met: metformin, Mela: Melatonin, Lpra: Ipragliflozin, Losa; losartan, Lira: liraglutide, L-Car: L-carnitine, Insu: insulin, Glicl: gliclazide

After selecting studies, 84 studies were included in the meta-analysis ALT outcome. Heterogeneity was not significant between studies (Q-value = 52.83, *P* value = 0.002, I-square = 55.9). Figure 3 shows the network of interventions introduced into the network meta-analysis for ALT outcome.

Based on the ratings given the therapeutic effect of ALT on the follow-up score, it is evident that treatment with Atorvastatin had the most significant impact on ALT compared to other investigated drugs. Table 3 shows the treatment ratings by use of P-score for ALT.

**Table 3:** Treatment ratings by use of P-score for ALT

**Rank**	**Drug**	**P-score (fixed)**	**P-score (random)**	**RANK**	**DRUG**	**P-score(fixed)**	**P-score(random)**
1	Atro	0.913	0.8787	20	Empa	0.5179	0.5189
2	Coles	0.8828	0.8622	21	Toco	0.4967	0.5027
3	Ezet	0.7976	0.8612	22	Prob	0.5459	0.5004
4	SOC	0.8719	0.8323	23	Pant	0.4512	0.4393
5	Glicl	0.8714	0.8273	24	Tryp	0.4256	0.4388
6	Sita	0.8243	0.7925	25	Sily	0.3156	0.3646
7	Plac	0.8153	0.7865	26	Obet	0.3073	0.3542
8	Feno	0.8006	0.7256	27	Bicy	0.2251	0.3061
9	Omeg3	0.773	0.7243	28	Piog	0.2948	0.2823
10	Insu	0.6554	0.6368	29	Beta	0.2371	0.2633
11	Telm	0.6556	0.6324	30	Lpra	0.2427	0.2578
12	UDCA	0.6985	0.6072	31	Exen	0.1934	0.2575
13	Losa	0.6211	0.6029	32	Synb	0.3278	0.2498
14	VitE	0.5289	0.5824	33	Orli	0.1487	0.2049
15	Mela	0.5741	0.5652	34	Vida	0.1102	0.1504
16	Resv	0.5831	0.5592	35	Rosi	0.0979	0.1464
17	Antiox	0.5688	0.5568	36	L-Carni	0.0875	0.1303
18	Lira	0.5249	0.5513	37	Probu	0.0002	0.0015
19	Met	0.5141	0.5462				

**Figure 4:**
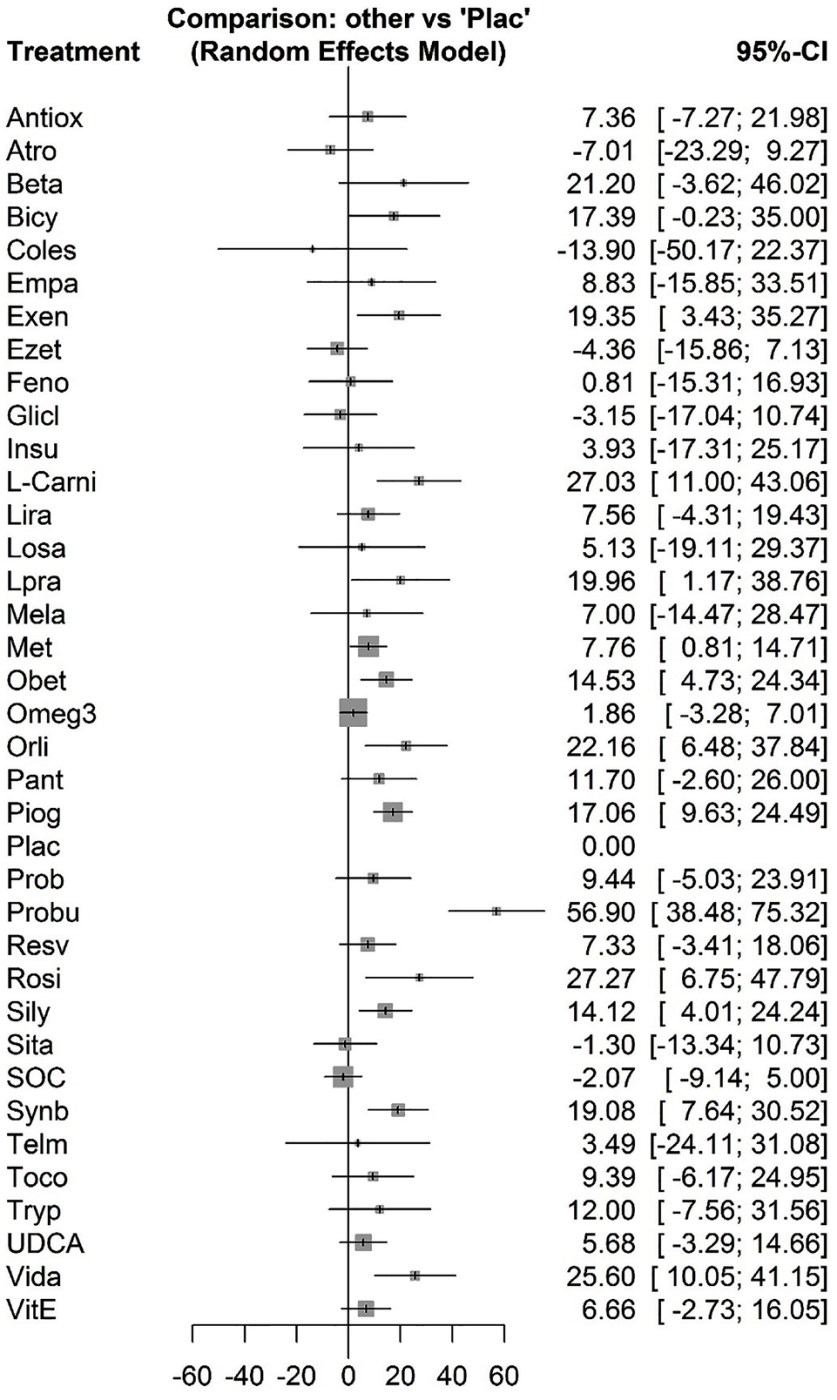
Combined results of netwrok meta-analysis with placebo.

The forest plot has also combined preliminary results with studies performed on the network meta-analysis in comparison to the placebo treatment (Figure 4). The level of evidence for the impact of atorvastatin vs. placebo on ALT levels has been calculated as low using GRADE approach.

#### Comparison of treatment effects on AST

After selecting studies, 71 cases were included in network meta-analysis for AST.

**Figure 5:**
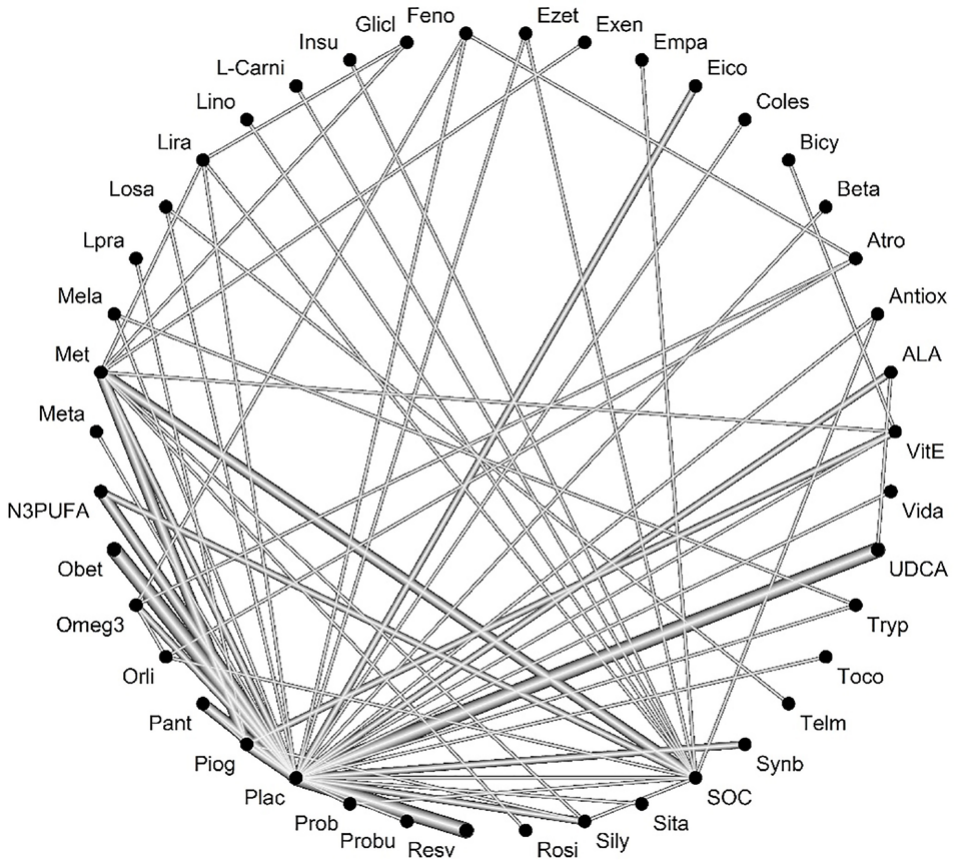
Network of interventions introduced into the network meta-analysis for AST outcome. Legend: Feno:fenofibrate, Ezet: ezetimbe, Exen: exenatide, Coles: Colesevelam, Bicy: bicycline, Beta:betaine, Atrp: atrovastatin, Antiox: anti-oxidants, Vit-E: vitamin- E, Vida:vidagliptin, UDCA: Ursodeoxycholic acid, Tryp: tryptophan, Toco: Tocotrienol, Telm: telmisartan, Synb:symbiotic, SOC: Standard of Care, Sita: sitagliptin, Silly:silymarin, Rosi: rosiglitazone, Resv: Resveratrol, Probu: probucol, Prob: probiotic, Plac:placebo, Piog: pioglitazone, Pant: Pentoxifylline, Orli: Orlistat, Omeg3: Omega-3, Obet: obeticholic acid, Met: metformin, Mela: Melatonin, Lpra: Ipragliflozin, Losa; losartan, Lira: liraglutide, L-Car: L-carnitine, Insu: insulin, Glicl: gliclazide

Heterogenicity was significant between studies (Q-value = 261.46, *P* value <0.001, I-square = 86.4). Figure 5 represents the network of interventions introduced into the network meta-analysis for the AST outcome. Based on the ratings given the therapeutic effect on AST, it was found that treatment with tryptophan had the best change over AST in comparison to the other investigated drugs.

Table 4 shows the treatment ratings by use of the P-score. The level of evidence for impact of tryptophan vs. placebo on AST levels has been calculated as low using GRADE approach.

**Table 4:** Treatment ratings by use of the P- score on AST

**Rank**	**Drug**	**P-score (fixed)**	**P-score (random)**	**Rank**	**Drug**	**P-score (fixed)**	**P-score (random)**
1	Tryp	0.9533	0.9181	20	Met	0.5205	0.516
2	Meta	0.952	0.9176	21	Rosi	0.5131	0.509
3	Coles	0.875	0.8413	22	Ezet	0.373	0.4945
4	Mela	0.8726	0.8144	23	Toco	0.4676	0.4892
5	Atro	0.8426	0.7784	24	Empa	0.3669	0.4191
6	Telm	0.799	0.7624	25	VitE	0.2622	0.3369
7	Sita	0.7806	0.7285	26	Prob	0.4346	0.3234
8	Losa	0.7278	0.7099	27	Lpra	0.2579	0.3122
9	Plac	0.682	0.6759	28	Piog	0.2909	0.3104
10	SOC	0.6417	0.667	29	Pant	0.265	0.3062
11	Glicl	0.8012	0.6637	30	Exen	0.2447	0.3046
12	UDCA	0.65	0.6577	31	Obet	0.178	0.2871
13	Feno	0.759	0.6347	32	Orli	0.1675	0.2749
14	Omeg3	0.6874	0.6301	33	Bicy	0.1107	0.2111
15	Resv	0.4763	0.6035	34	Synb	0.3962	0.2061
16	Antiox	0.669	0.5915	35	Beta	0.1376	0.1852
17	Insu	0.5092	0.5364	36	L-Carni	0.0808	0.1574
18	Lira	0.7011	0.5336	37	Vida	0.0847	0.1555
19	Sily	0.4669	0.5254	38	Probu	0.0015	0.011

#### Network meta-analysis results for secondary outcomes

#### Comparison of the effects of the treatment on BMI

After selecting the studies, 48 cases were included in the meta-analysis. Heterogeneity was not significant between studies (Q-value = 15.98, *P* value = 0.31, I-square = 0.00).

**Figure 6:**
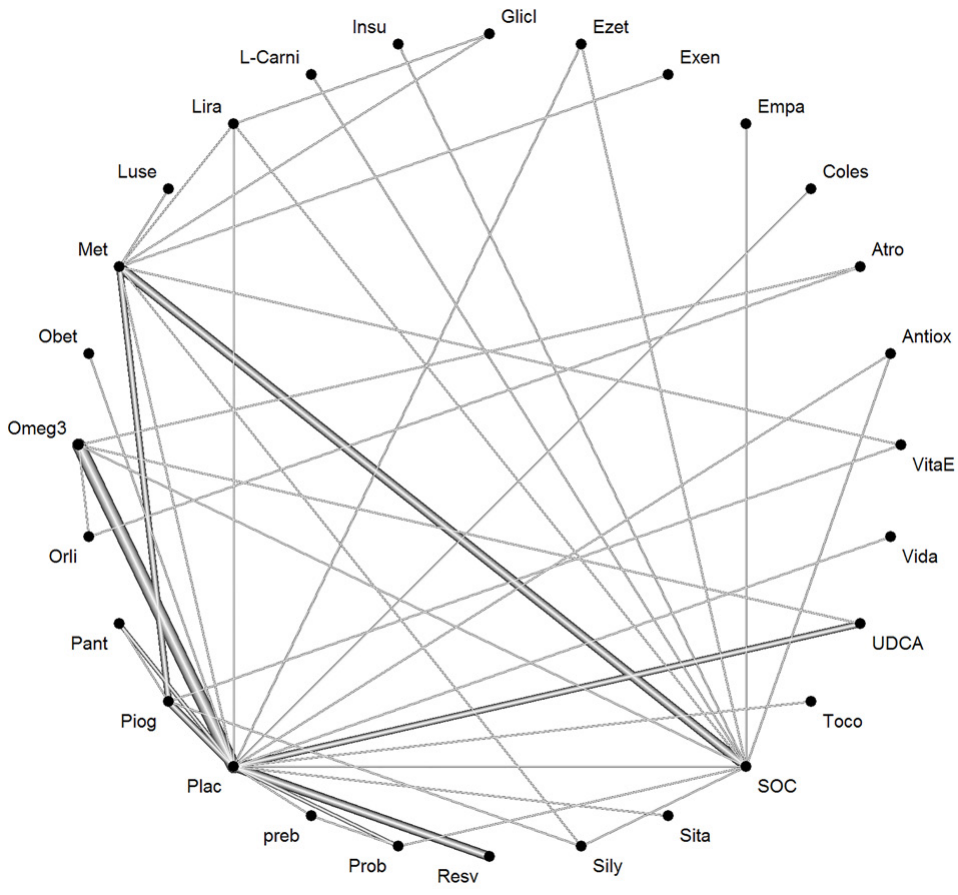
Network of interventions introduced into the network meta-analysis for BMIoutcome.
Legend: Feno:fenofibrate, Ezet: ezetimbe, Exen: exenatide, Coles: Colesevelam, Bicy: bicycline, Beta:betaine, Atrp: atrovastatin, Antiox: anti-oxidants, Vit-E: vitamin- E, Vida:vidagliptin, UDCA: Ursodeoxycholic acid, Tryp: tryptophan, Toco: Tocotrienol, Telm: telmisartan, Synb:symbiotic, SOC: Standard of Care, Sita: sitagliptin, Silly:silymarin, Rosi: rosiglitazone, Resv: Resveratrol, Probu: probucol, Prob: probiotic, Plac:placebo, Piog: pioglitazone, Pant: Pentoxifylline, Orli: Orlistat, Omeg3: Omega-3, Obet: obeticholic acid, Met: metformin, Mela: Melatonin, Lpra: Ipragliflozin, Losa; losartan, Lira: liraglutide, L-Car: L-carnitine, Insu: insulin, Glicl: gliclazide

The network of interventions introduced into the network meta-analysis for BMI outcomes is demonstrated in Figure 6.

Based on the given ratings related to therapeutic effects on BMI, it was found that treatment with Orlistat had the best efficiency in BMI over the other drugs studied. Table 5 shows the treatment ratings by use of the P-score. The level of evidence for the impact of orlistat vs. placebo on BMI has been calculated as low using GRADE approach.

**Table 5:** Treatment ratings by use of the P-score

**Rank**	**Drug**	**P-score (fixed)**	**P-score (random)**	**Rank**	**Drug**	**P-score (fixed)**	**P-score(random)**
1	Orli	0.989	0.989	15	preb	0.4783	0.4783
2	Vida	0.892	0.892	16	Obet	0.4507	0.4507
3	Exen	0.8776	0.8776	17	Sily	0.4427	0.4427
4	Antiox	0.8236	0.8236	18	Omeg3	0.401	0.401
5	Empa	0.7142	0.7142	19	Piog	0.384	0.384
6	Lira	0.7093	0.7093	20	Coles	0.3758	0.3758
7	Insu	0.6821	0.6821	21	Toco	0.3401	0.3401
8	L-Carni	0.6109	0.6109	22	VitaE	0.3401	0.3401
9	Ezet	0.6033	0.6033	23	Atro	0.3106	0.3106
10	Prob	0.5963	0.5963	24	UDCA	0.2293	0.2293
11	SOC	0.5942	0.5942	25	Resv	0.2165	0.2165
12	Luse	0.5689	0.5689	26	Sita	0.1755	0.1755
13	Met	0.5285	0.5285	27	Plac	0.1516	0.1516
14	Pant	0.4962	0.4962	28	Glicl	0.0178	0.0178

#### Comparison of the effects of the treatment on steatosis

After selecting the studies, 14 studies were included in the meta-analysis. Heterogenicity was not significant between studies (Q-value = 0.43, *P* value = 0.51, I-square = 0.00).

**Figure 7:**
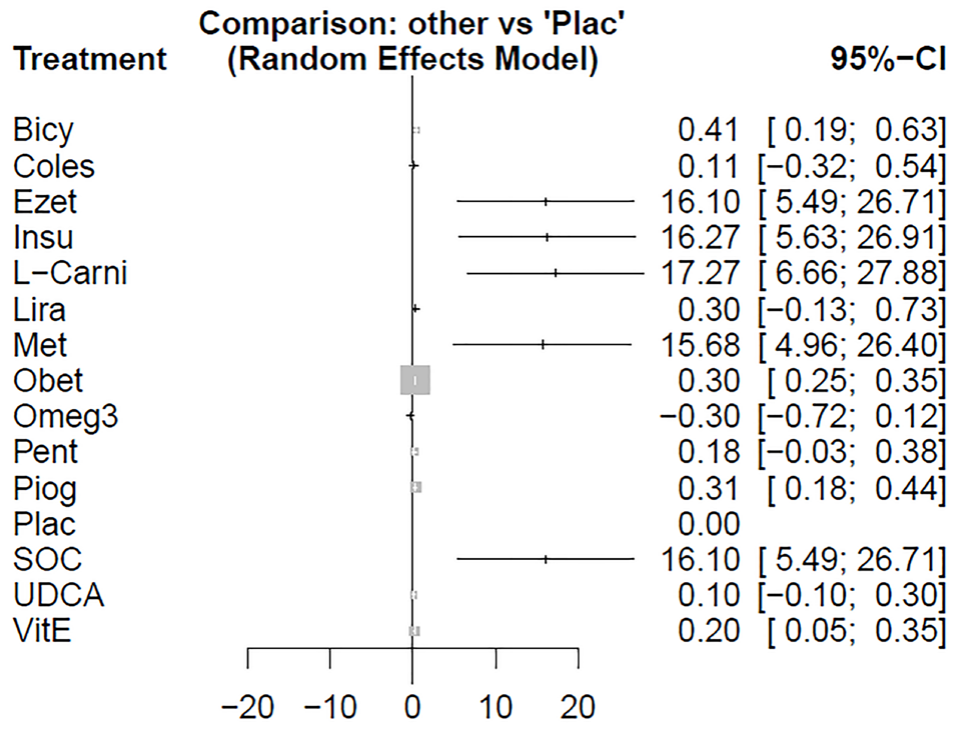
Forest plot of comparison of medication for steatosis.
Beta:betaine, Atrp: atrovastatin, Antiox: anti-oxidants, Vit-E: vitamin- E, Vida: vidagliptin, UDCA: Ursodeoxycholic acid, Tryp: tryptophan, Toco: Tocotrienol, Telm: telmisartan, Synb:symbiotic, SOC: Standard of Care, Sita: sitagliptin, Silly:silymarin, Rosi: rosiglitazone, Resv: Resveratrol, Probu: probucol, Prob: probiotic, Plac:placebo, Piog: pioglitazone, Pant: Pentoxifylline, Orli: Orlistat, Omeg3: Omega-3, Obet: obeticholic acid, Met: metformin, Mela: Melatonin, Lpra: Ipragliflozin, Losa; losartan, Lira: liraglutide, L-Car: L-carnitine, Insu: insulin, Glicl: gliclazide

Based on the given ratings associated with the therapeutic effects of steatosis, it was concluded that treatment with Omega-3 had the best efficiency over steatosis in comparison to the other investigated drugs. Table 6 shows the ratings of treatments using the P-score. The forest plot has also been shown to combine preliminary results with studies performed on the meta-analysis of networks compared with placebo treatment (Figure 7). The level of evidence for impact of Omega-3 vs. placebo on steatosis has been calculated as low using GRADE approach.

**Table 6:** Ratings of treatments using the P- score for steatosis

**Rank**	**Intervention**	**P-score (fixed)**	**P-score (random)**
1	Omeg3	0.979	0.979
2	Plac	0.8906	0.8906
3	UDCA	0.7794	0.7794
4	Coles	0.7403	0.7403
5	Pent	0.6891	0.6891
6	VitE	0.6728	0.6728
7	Lira	0.5653	0.5653
8	Obet	0.5233	0.5233
9	Piog	0.5173	0.5173
10	Bicy	0.4231	0.4231
11	Met	0.2247	0.2247
12	Ezet	0.1765	0.1765
13	SOC	0.1765	0.1765
14	Insu	0.1389	0.1389
15	L-Carni	0.0033	0.0033

#### Comparison of the treatment effects on the NAS

After selecting studies, 15 cases were included in the meta-analysis. Heterogeneity was not significant between studies (Q-value = 1.15, *P* value = 0.28, I-square = 12.8).

**Figure 8:**
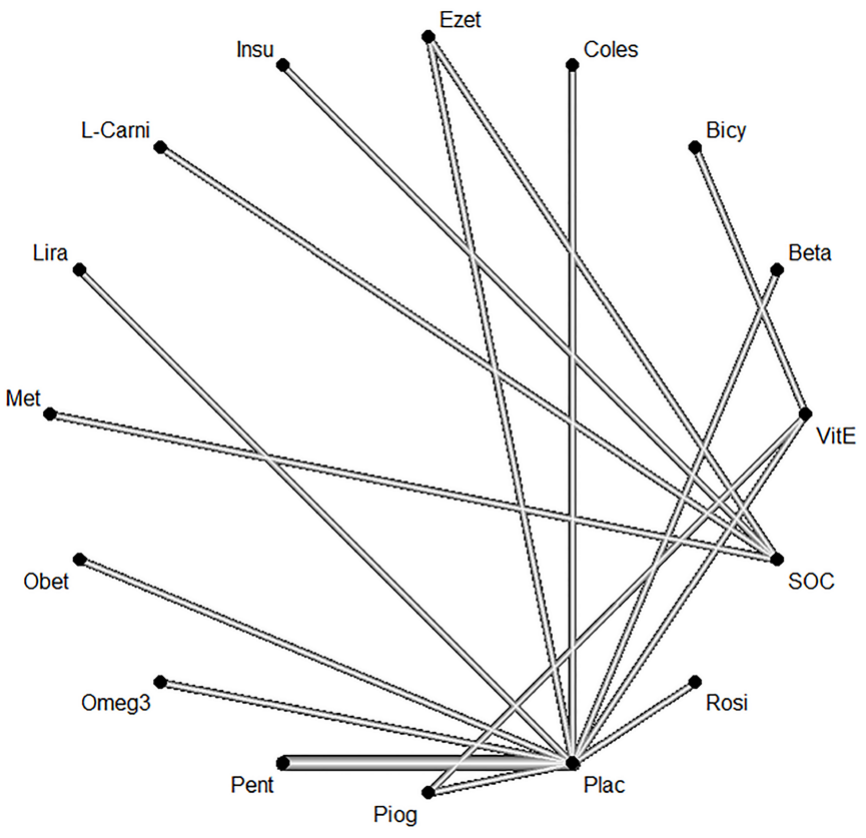
Network of interventions introduced into the network meta-analysis for NAS
Legend: Feno:fenofibrate, Ezet: ezetimbe, Exen: exenatide, Coles: Colesevelam, Bicy: bicyclineBeta:betaine, Atrp: atrovastatin, Antiox: anti-oxidants, Vit-E: vitamin- E, Vida:vidagliptin, UDCA: Ursodeoxycholic acid, Tryp: tryptophan, Toco: Tocotrienol, Telm: telmisartan, Synb:symbiotic, SOC: Standard of Care, Sita: sitagliptin, Silly:silymarin, Rosi: rosiglitazone, Resv: Resveratrol, Probu: probucol, Prob: probiotic, Plac:placebo, Piog: pioglitazone, Pant: Pentoxifylline, Orli: Orlistat, Omeg3: Omega-3, Obet: obeticholic acid, Met: metformin, Mela: Melatonin, Lpra: Ipragliflozin, Losa; losartan, Lira: liraglutide, L-Car: L-carnitine, Insu: insulin, Glicl: gliclazide

The network of interventions introduced into the network meta-analysis for the outcome of NAS are shown in Figure 8.

**Figure 9:**
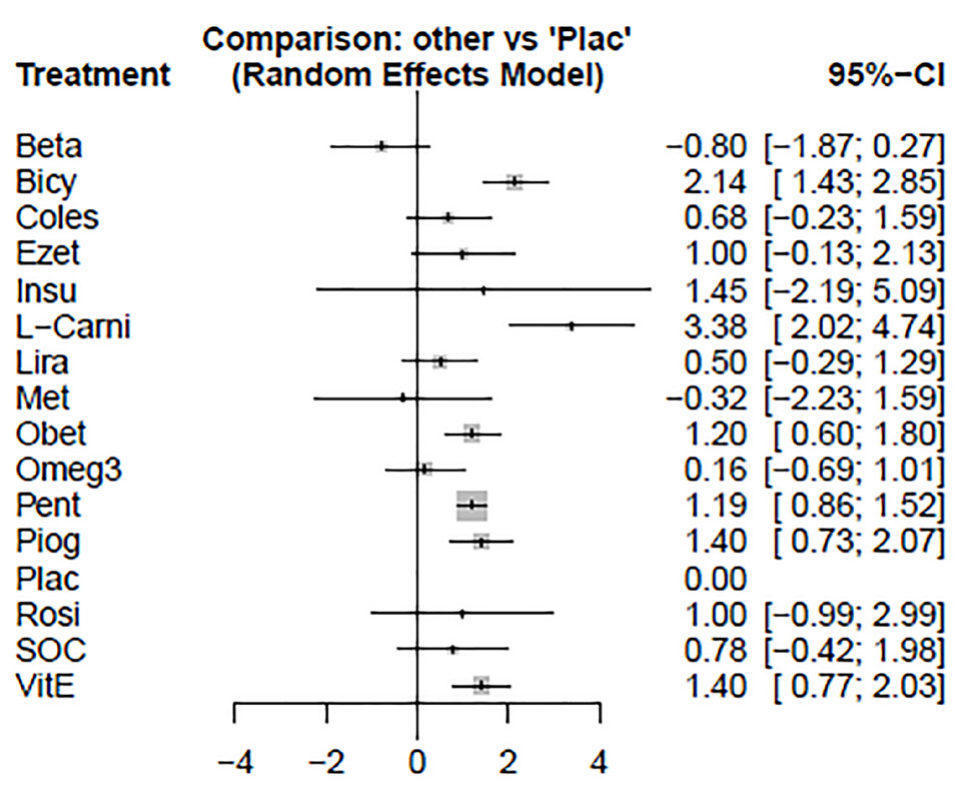
Preliminary results with studies performed on the meta-analysis of networks compared with placebo treatment on NAS.
Legend: Feno:fenofibrate, Ezet: ezetimbe, Exen: exenatide, Coles: Colesevelam, Bicy: bicycline, Beta:betaine, Atrp: atrovastatin, Antiox: anti-oxidants, Vit-E: vitamin- E, Vida:vidagliptin, UDCA: Ursodeoxycholic acid, Tryp: tryptophan, Toco: Tocotrienol, Telm: telmisartan, Synb:symbiotic, SOC: Standard of Care, Sita: sitagliptin, Silly:silymarin, Rosi: rosiglitazone, Resv: Resveratrol, Probu: probucol, Prob: probiotic, Plac:placebo, Piog: pioglitazone, Pant: Pentoxifylline, Orli: Orlistat, Omeg3: Omega-3, Obet: obeticholic acid, Met: metformin, Mela: Melatonin, Lpra: Ipragliflozin, Losa; losartan, Lira: liraglutide, L-Car: L-carnitine, Insu: insulin, Glicl: gliclazide.

Based on the given ratings pertinent to the therapeutic effects on NAS, it was observed that treatment with betaine had the best efficiency over NAS compared to the other investigated drugs. The forest plot has also been shown to combine preliminary results with studies performed on the meta-analysis of networks compared with placebo treatment (Figure 9). The level of evidence for impact of betaine vs. placebo on NAS has been calculated as low using GRADE approach (Table 7).

**Table 7:** The level of evidence for each first identified intervention on primary and secondary outcomes vs. placebo

**Certainty assessment**							**Effect**		**Certainty**
**Outcome**	**Study design**	**Risk of bias**	**Inconsistency**	**Indirectness**	**Imprecision**	**Other considerations**	**Relative (95% CI)**	**Absolute (95% CI)**	
ALT(Atro vs Plac)	Randomized trials	Not serious	Serious	Serious	Not serious	None	-	MD **7.01** (23.22 to 9.27)	⨁⨁OO LOW
AST (Tryp vs Plac)	Randomized trials	Not serious	Serious	Serious	Not serious	None	-	**16** (34.11 to 2.11)	⨁⨁OO LOW
BMI (Orli vs Plac)	Randomized trials	Not serious	Serious	Serious	Not serious	None	-	**4.47** (5.91 to 3.02)	⨁⨁OO LOW
Steatosis (Omeg3 vs Plac)	Randomized trials	Not serious	Serious	Serious	Not serious	None	-	MD **0.3** (0.72 to 0.12)	⨁⨁OO LOW
NAS (Met vs Plac)	Randomized trials	Not serious	Serious	Serious	Not serious	None	-	MD **0.32** (2.23 lower to 1.59 higher)	⨁⨁OO LOW

CI: Confidence interval; MD: Mean difference

Based on our search of medical databases and although there is a meta-analysis conducted on the topic of effective medication on NAFLD conducted by Sridharan et al our research is the first network meta-analysis that has been conducted on this topic among adult population so far. Additionally, our systematic review and network meta-analysis is different than the previous work as this article has also included children’s studies that could potentially have confounding factors as NAFLD in children ma arise from genetic origins.^103^ Another major difference that is noted in the conduct of the present stud and the research by Sridharan et.al, is that despite our study, authors in this comparable study has different set of primary and secondary outcomes. As mentioned in the methods and materials section, we have set our primary outcomes for changes in ALT, AST and secondary outcomes include change in BMI, NAS and hepatic steatosis whereas, Sridharan and colleagues has put overall response rate to medication and AST, ALT, NAS, BMI, lipid profiles were set as secondary outcome.

We have implicated the primary impact of different medical therapies and their effect on liver transaminases, as our primary outcome of NAFLD. We showed that atorvastatin, colesevelam and ezetimibe had the best outcome compared to the SOC on ALT, and tryptophan and melatonine had the best impact on lowering AST while in the study of Sridharan and colleagues, the best medication to reduce ALT is reported to be with vitamin D followed by gemfibrozil and ipragliflozin.^104^

Based on the given ratings pertinent to the therapeutic effects on NAS, it was observed that treatment with Betaine had the best efficiency over NAS compared to the other investigated drugs.

Sridharan et al have determined the better efficacy for elafibranor.^104^ While Sridharan et al had reported the best outcome from telmisartan, in the current investigation, Telmisartan had lower efficacy than SOC on.^104^

High-quality clinical trials are needed to prove the efficacy of atorvastatin in NAFLD.

As we expected, life-style modification shown to be effective in reducing aminotransferases. Alongside the use of medicinal compound. Of these, orlistat was the first effective drug followed by liraglutide. Given to the fact that treatment of NAFLD can be associated with complications including NAS, we illustrated that betaine, metformin, omega 3, liraglutide and colesevelam were most effective drugs in reduction of NAS. Of these, efficacy of betaine and liraglutide had similarities to the results of previous meta-analyses. Omega 3 were shown to be best therapeutic choice in hepatic steatosis.

In previous meta-analysis, pentoxifylline was one of the drugs that had a remarkable effect on reducing liver fibrosis. Compared to our findings, although pentoxifylline were a weaker agent in reducing NAS, it worked well.^104^

According to the guidelines of American Association for Liver Diseases, vitamin E administered at a daily dose of 800 IU/day improves liver histology in nondiabetic adults with biopsy-proven NASH and therefore may be considered for this patient population.^9^

Based on the results of our meta-analysis, vitamin E had no significant impact on the reduction of aminotransferases compared to SOC in the context of decline hepatic steatosis, nevertheless, has not been shown to be effective in reducing NAS.

We observed no obvious effect for polyunsaturated fatty acids (PUFA) for both primary and secondary outcomes, therefore, its administration in NAFLD is not recommended.

Ultimately for NAS, our meta-analysis revealed the best response from Betaine. It is also recommended to include the betaine in larger clinical trials to further study its anti-steatotic properties.

We encountered some limitation in performing current systematic review, heterogenicity of studies and the lack of studies that had investigated the combined medications are some of them.

It is suggested to do other meta-analyses to update previous studies, which had reduced the amount of drug diversity. A general consensus about some drugs and patient’s treatment process is needed in prospective studies as well.

## Conclusion

All in all, our study shows a higher efficacy for the reduction in liver transaminases for atorvastatin. Although lifestyle modification, weight loss and BMI reduction are all effective in improving the primary and secondary outcomes, our network meta-analysis showed the greater efficacy for tryptophan, orlistat, omega-3 and betaine to improve AST, BMI, steatosis and NAS. Considering these findings, we recommend randomized clinical trials to examine these medical modalities with placebo and each other.

## Ethical Issues

This systematic review and meta-analysis has been evaluated and approved by regional ethics at Tabriz university of Medical Sciences, Faculty of Medicine. Additionally, all ethical considerations were meet in all levels of systematic search according to systematic review and meta-analysis

## Conflict of Interests

None to declare.

## Acknowledgments

This research was financially supported by the Tabriz University of Medical Sciences, Tabriz, Iran. The authors would like to thank the Tabriz University of Medical Sciences for providing the expertise that greatly assisted.

## Supplementary Materials

Supplementary file 1 contains Table S1.
