# Supplementary material for: The Evaluation of Effective Drugs for the Treatment of Non-Alcoholic Fatty Liver Disease: A Systematic Review and Network Meta-Analysis
Source: Adv Pharm Bull. 2020 Aug 9;10(4):542–55. doi: 10.34172/apb.2020.065 (PMC7539311; doi:10.34172/apb.2020.065)
Supplement: Supplementary file 1 — contains Table S1. [file apb-10-542-s001.pdf]

Supplementary file 1

Table S1. Data extraction table

Author	Country	Duration	Type of Group	Type of Treat meant			sample size			Age		
				Group1	Group2	Group3	Group1	Group2	Group3	Group1	Group2	Group 3
Marchesini et al (2001)[18]	Italy	4 M	NASH	Metformin 1500 mg/day	Diet		14	6		40	40	
Athyros et al (2006)[19]	Greece	54 W	NAFLD(metabolic syndrome)	Atorvastatin, 20 mg/day	Fenofibrate, 200 mg/day		63	62		60	61	
Ratziu et al (2008)[20]	France	1 Y	NASH	rosiglitazone (8mg/day)	placebo		32	31		53.1	54.1	
Hatzitolios A. et al (2004)[21]	Greece	24 W	NAFLD with dyslipidemia	Omega3 5ml t.i.d.	Atorvastatin 20mg	Orlistat 120 mg t.i.d.	23	28	21	52	53	50
Fan H. et al (2013)[22]	China	12 W	T2 diabetes with NAFLD	Exenatide injection 10mcg b.i.d.	metformin 2g/day		49	68		51.02	54.68	
CAPANNI M. et al (2006)[23]	Italy	12 M	NAFLD	Placebo	n-3 PUFA		14	42		56	58	
Solhi H. et al (2014)[24]	Iran	8 W	NASH	silymarin	placebo		33	31		43.6	39.36	
Khoshbaten M. et al (2010)[25]	Iran	3 M	NAFLD	N-acetylcysteine	Vit C		15	15		40.1	46.8	
Lindor K. et al (2004)[26]	Canada, US	2 Y	NASH	13 to 15 mg/kg/d of oral UDCA	Placebo		80	86		45.4	48.5	
Zein C. et al (2011)[27]	USA	1 Y	NASH	pentoxifylline 400 mg t.i.d.	placebo		26	29		50.5	49.6	

Muhammad Amjad Pervez (2017)[28]		12M	NAFLD	Tocotrienol	PLACEBO		31	33		44.7	43.9	
K. Shahebrahimi (2017)[29]			NAFLD	two gr/day metformin	30 mg/day pioglitazon e	800 IU/D VITAMIN E	31	31	31	43.87	43.87	43.87
Chan Wah Kheong (2017)[30]	Malaysia	22 M	NAFLD	silymarin	placebo		49	50		49.6	50.1	
Jan Oscarsson (2018)[31]	Sweden	3 M	hypertriglyceride mia OR NAFLD	Placebo	Omega-3	Fenofibrat e	26	25	27	59.5	60	64
Daisuke Ito (2017)[32]	Japan	13 M	NAFLD AND DM-T2	Pioglitazone group	Ipragliflozi n group		34	32		59.1	57.3	
Somayyeh Asghari (2018)[33]	Iran	3 M	NAFLD	placebo	Resveratro l		30	30		38.5	40	
K. CELINSKI (2014)[34]	POLAND	14 M	NAFLD	tryptophan	melatonin	placebo	28	23	23			
Srinivasan Dasarathy, MD (2015)[35]	USA	12 M	NAFLD AND DM	omega 3 fatty acids	placebo (CORN OIL)		18	19		51.5	49.8	
Abdelmalek (2009)[36]	usa	27 M	NAFLD	Betaine	Placebo		27	28		47.8	45.7	
Alam (2016)[37]	Italy	34 M	NAFLD	Telmisartan	underwent lifestyle modificati on alone		20	10		43.3	37.9	
ALLER (2011)[38]	Spain	3 M	NAFLD	probiotic	placebo		14	14				
Argo (2015)[39]	USA	3 Y	NAFLD	Placebo	N-3 PUFA		17	17		47.2	46.4	
Armstrong(2016)[40]	UK	48 W	non-alcoholic steatohepatitis	liraglutide	Placebo		26	26		50	52	
Balas (2007)[41]	USA	4 W	impaired glucose tolerance or T2DM and NASH	pioglitazone	Placebo		21	14		46.4	51	

Baniasadi (2015)[42]	IRAN	6 M	NASH	pantoxifyllin	Placebo Group		15	15		35.7	39.4	
Belfort (2006)[43]	USA	6 M	nonalcoholic steatohepatitis	Placebo	Pioglitazon e		21	26		51	51	
Chachay (2014)[44]	Australia	8 W	NAFLD	resveratrol	PLACEBO		10	10		48.8	47.5	
Chen (2015)[45]	China	3 M	NAFLD	Placebo	Resveratro l		30	30		43.5	45.2	
Cui (2016)[46]	USA	6 M	hepatic steatosis	sitagliptin	placebo		25	25		52.9	54.9	
Cusi (2016)[47]	USA	6 Y	NASH and prediabetes or T2DM	Placebo	Pioglitazon e		51	50		49	52	
Deng (2017)[48]	China	3 Y	NAFLD AND T2DM	Sitagliptin	Diet		36	36		63.7	64.1	
Ebrahimi-Mameghani (2016)[49]	Iran	15 M	NAFLD	DIET	linoleic acid		19	19		36.74	38.58	
Ekhlasi (2017)[50]	Iran	3 M	NAFLD	Probiotic	Placebo		32	32		12.7	12.6	
Eslamparast (2014)[51]	Iran	28 W	NAFLD	Synbiotic	Placebo		26	26		46.35	45.69	
Faghihzadeh (2014)[52]	Iran	12 W	NAFLD	Resveratrol	PLACEBO		25	25		44.04	46.28	
Feng (2017)[53]	China	6 M	T2DM with NAFLD	Liraglutide	Gliclazide	Metformin	29	29	29	46.79	48.07	46.31
Garinis (2010)[54]	Italy	6 M	NAFLD	Metformin	Diet		20	25		40.8	45.8	
Hajiaghamohammadi(2012)[55]	Iran	4 M	NAFLD	Pioglitazone	Metformin	Silymarin	22	22	22	32.62	32.62	32.62
Han (2014)[56]	China	6 M	NAFLD	METFORMIN	Vitamin E		112	111				
Harrison (2003)[57]	USA	6 M	Nonalcoholic Steatohepatitis	Placebo	Vitamin E,VIT.C		22	23		50.2	52.5	
Harrison (2009)[58]	USA	24 M	Nonalcoholic Steatohepatitis	DIET Group	Orlistat Group		18	23		45.8	47.9	

Haukeland(2009)[59]	Norway	6 M	NAFLD	Placebo	Metformin		24	20		49.9	44.3	
Heebøll (2016)[60]	Denmar k	6 M	NAFLD	Resveratrol	PLACEBO		13	13				
Hirata (2013)[61]	Japan	12 M	NAFLD and hypertension	Telmisartan	Losartan		12	7		57.7	60.3	
Hussain (2016)[62]	Pakistan	12 W	NAFLD	Vildagliptin	Placebo		29	29		28	31	
Khoo (2017)[63]	Singapor e	21 M	NAFLD	diet	Liraglutide		12	12		43.7	39	
LE (2012)[64]	USA	24 W	NASH	Colesevelam	Placebo		25	25		45.4	50.3	
Lee (2008)[65]	Singapor e	3 M	NASH	Pentoxifylline	PLACEBO		11	9		47	47.89	
Leuschner (2010)[66]	German y AND Greece	18 M	NASH	UCDA	PLACEBO		95	91		41.45	45.02	
Li (2015)[67]	China	6 M	NASH	PLACEBO	PUFA		39	39		50.4	52.6	
Loomba (2015)[68]	USA	24 W	NASH	Ezetimibe	Placebo		25	25		49	49.5	
McPherson (2017)[69]	UK	2 Y	NAFLD	Losartan	PLACEBO		24	21		58	45	
Mendez-Sanchez (2004)[70]	Mexico	6 W	NAFLD	UCDA	PLACEBO		12	11		39.7	37.8	
Merat (2003)[71]	Iran	6 M	NASH	drug:probuco l	PLACEBO		18	9		36.3	35.9	
Mofidi (2017)[72]	Iran	28 W	NAFLD	synbiotic supplementa tion	placebo		21	21		40.9	44.61	
van Wagner (2011)[73]	USA Chicago, IL	3 Y	NAFLD	PANTOXYPHY LINE 400 mg	placebo		21	9		48	53	
Zelber-Sagi (2006)[74]	Israel	2 M	NAFLD	orlistat 120 mg	placebo		21	23		48.4	47	
Wah Kheong (2017)[75]	Malaysia	2Y	NAFLD	silymarin 700 mg	placebo		49	50		49.6	50.1	
Wong (2013)[76]	China	6M	NAFLD	probiotic	DIATE		10	10		42	55	
Tiikkainen (2004)[77]	Finland	16 W	NAFLD	Rosiglitazone	Metformin		9	11		50	46	

Zhu (2008)[78]	China	24 W	NAFLD	n-3 PUFA	placebo		66	68		45	44.03	
Mudaliar (2013)[79]	USA	6 W	NAFLD AND T2DM	Placebo	25 mg OBETICHOLIC ACID	50 mgOBETICHOLIC ACID	23	20	21	53.1	52.7	50.5
Nelson (2009)[80]	USA	72 W	FLINT	Obeticholic acid	PLACEBO		141	142		52	51	
Nogueira (2016)[81]	Brazil	6 M	NASH	OMEGA - 3	PLACEBO		27	23		53.9	52.5	
Omer (2009-10)[82]	Turkey	1 Y	NAFLD	metaformin	Rosiglitazone	METformin+ROSIgiltazoe	22	20	22	48	49.3	49.6
PAKRAVAN(2017)[83]	Iran	3 M	NAFLD	Melatonin	PLACEBO		47	48		42.5	40.6	
Parikh (2016)[84]	India	52 W	NAFLD	(Vitamin E)	(UDCA)		95	138		43.75	40.19	
Ratziu (2016)[85]	USA	52 W	NAFLD	Placebo	Elafibranor, 80 mg	Elafibranor, 120 mg	92	93	89	52.4	52.7	52.4
Razavizade(2013)[86]	Iran	4 M	NAFLD	Metformin	Pioglitazone		40	40		36.35	34.2	
Santos (2003)[87]	Brazil	3 M	NAFLD	UDCA	PLACEBO		15	15		38.4	36.6	
SANYAL (2010)[88]	USA	96 W	NASH	Placebo	Vitamin E	Pioglitazone	83	84	80	45.4	46.6	47
SANYAL (2014)[89]	USA	2 Y	NASH	Placebo	EPA-E(eicosapentanoic) (1800 mg/d)	EPA-E(eicosapentanoic (n ¼ 2700 mg/d	72	82	86	50.5	47.8	47.8
Sharma (2012)[90]	India	6 M	NASH	Pentoxifylline	Pioglitazone		11	9		37.3	40.4	
Qin (2015)[91]	China	3 M	NAFLD	CORN OIL	FISH OIL		34	36		44.3	46	
Shibuya (2017)[92]	Japan	3 M	NAFLD	Luseogliflozin	Metformin		16	16		51	60	
Takeshita (2014)[93]	Japan	6 M	NAFLD	DIET	Ezetimibe		14	17		55.5	50.4	
Sofi (2010)[94]	Italy	12 M	NAFLD	N3 PUFA	diet		6	5		55	54	
Chen (2008)[95]	China	24 W	NAFLD or NASH	Placebo	Omega-3 8cap/day	Omega-3 10cap/day	16	15	15	45	47	46

Shenoy (2014)[96]	Italy	16 W	NASH	Metadoxine 500mg/day	Placebo		75	59		39.8	41.1	
Hameed (2017)[97]	USA	96 W	NASH	Obeticholic acid+weight loss	Placebo+weight loss		102	98		52	50	
Amirkhizi (2018)[98]	Iran	12 W	NAFLD patients with obesity	ALA	Placebo		23	22		40.6	38.8	
Shafi Kuchay (2018)[99]	India	20 W	NAFLD Patients With Type 2 Diabetes	Empagliflozin	diet		22	20				
Sofer (2011)[100]	Israel	16 W	NAFLD	Metformin	Placebo		32	31		51.9	55.2	
Abenavoli (2017)[101]	Italy	24 W	NAFLD	Mediterranean diet	without drug		20	10		52	33	
